# Cancer and the healthy immigrant effect: a statistical analysis of cancer diagnosis using a linked Census-cancer registry administrative database

**DOI:** 10.1186/s12889-017-4190-2

**Published:** 2017-04-05

**Authors:** James Ted McDonald, Michael Farnworth, Zikuan Liu

**Affiliations:** 1 Department of Economics, University of New Brunswick, PO Box 4400, Fredericton, NB E3B 5A3 Canada; 2 NB-Institute for Research, Data and Training, University of New Brunswick, PO Box 4400, Fredericton, NB E3B 5A3 Canada

**Keywords:** Immigration, Cancer, Socioeconomic status, Administrative data, Healthy immigrant effect

## Abstract

**Background:**

A large volume of research has been published on both the socio economic and demographic determinants of cancer and on the health of immigrants and minority groups. Yet because of data limitations, little research examines differences in the occurrence of cancer incidence between immigrants and non-immigrants and among immigrants defined by region of birth and time in the host country. In particular it is not known whether a healthy immigrant effect is present for cancer and if so, whether this advantage is lost with additional years of residence in the host country.

**Methods:**

This paper uses a large data file from Statistics Canada that links Census information on immigrant status, socioeconomic status including educational attainment, and other person-level information with administrative data on cancer and mortality over a continuous 13 year period of observation. It estimates discrete and continuous time duration models to identify differences in cancer diagnosis by immigrant subgroup after controlling for a variety of potential confounders. Differences in historical smoking behavior are not observable at the individual level in the dataset but are accounted for indirectly using various methods.

**Results:**

Results in general confirm the existence of a healthy immigrant effect for cancer in that, overall, recent immigrants to Canada are significantly less likely than otherwise comparable non-immigrant Canadians to be diagnosed with any cancer and the most common forms of cancer by site. As well, this gap appears to decline with additional years in Canada for immigrant men and women, eventually converging to Canadian-born levels. Differentiating among immigrant subgroups by period of arrival and country of birth reveals significant variation across immigrant subgroups, with immigrant men and women from developing countries typically having a lower likelihood of being diagnosed with cancer than immigrants from the US, UK and continental Europe. As well, controlling for immigrant heterogeneity this way weakens the conclusion that the gap narrows with years in Canada. Immigrant men overall continue to exhibit convergence to Canadian-born levels for diagnosis of any cancer and for prostate cancer, while immigrant women exhibit narrowing over time only for breast cancer. Although smoking behavior is not directly observed, controlling for subgroup-specific lifetime smoking behavior using survey data has only a relatively minor effect on the estimated differences.

**Conclusions:**

The specificity of the results by cancer type, gender, immigrant status and ethnicity provides useful guidance for future research by helping to narrow the possible channels through which social and economic characteristics may be affecting cancer incidence.

## Background

A large volume of research has considered the role of both demographic factors such as race or ethnicity and contextual factors such as socioeconomic status and place of residence in affecting the incidence of cancer. Depending on the cancer type, only 2–10% of cancers have been estimated to be the result of a mutation in, or the operation of, a particular gene (Lee Davis, Donovan, et al. [19]). This suggests that the overwhelming majority of cancer-causing factors are linked to individual behaviour, environmental context, or interactions between genes and these other contextual factors. Identifying and evaluating systematic differences in cancer incidence by socioeconomic status or geographic region of residence can advance our understanding of cancer and so help public health agencies to design policies for those at greater risk of the disease. Socioeconomic factors such as education and income have long been important correlates of cancer incidence and survival. Socioeconomic status may reflect a range of possible cancer determinants that are often not observed in administrative data sources, including health behaviors such as smoking, diet and activity, timely use of preventative health services, cancer screening, delayed childbirth, and exposure to carcinogens through occupational exposures (Link and Phelan [22]; Aronson et al. [3], [8] Lightfoot and Berriault [21]). Demographic and socioeconomic inequalities in cancer incidence persist even with improved knowledge of risk factors and improvements in early detection and treatment, (Clegg et al. [7]).

Much of this work has been limited in terms of available data in one or more dimensions. Data collected as part of a case control study are typically small in scope, which limits generalizability as well as the types of statistical analyses that can be conducted [4]. Health survey data reports information on personal demographic and socioeconomic characteristics however the number of cancer cases among immigrant or minority groups is relatively small and the occurrence of cancer is self-reported. Data drawn from national or state registries are usually limited to basic demographic information on age, sex and location of residence which means that information on many individual characteristics such as education, immigrant status and personal or household income is not available. As well, by their nature Cancer Registries contain data only on those diagnosed with cancer and not the underlying at-risk population. Underlying population is approximated using population counts by defined geographic area usually drawn from a population census. This necessitates that socioeconomic status must be measured using area-level data on median household income or similarly constructed variables. Unlike its Canadian counterpart, the US SEER databases contain data on race and some state registries report information on country of birth, though sometimes specific place of birth and immigrant status has been imputed from information such as patient’s name and age when a social insurance number is issued ([11, 24]). Cancer incidence and outcomes vary significantly by race and ethnicity in the US literature, and in particular overall cancer incidence rates are lower among Asians and Pacific Islanders than among non-Hispanic whites [44]. Newman [30] finds that while black women have a lower incidence of all-stage breast cancer, they also experience higher mortality rates (see also [27]). Since race/ethnicity is not recorded in Canadian Cancer Registry data, Hislop et al. [14] and Bashash et al. [5] impute ethnicity based on a review of patient names in the British Columbia Cancer Registry. Clegg et al. [6] compares race, Hispanic status and immigrant status between registry information and self- reports using the SEER--National Longitudinal Mortality Study linked database and found that while the degree of agreement was excellent for race and Hispanic status, it was low for immigrant status.

These limitations have encouraged interest in and development of linked individual-level datasets that combine administrative, survey and/or census information. Clegg et al. (2009) use the US SEER-NLMS dataset that links state cancer registry data to the National Longitudinal Mortality Study, a component of the Current Population Surveys linked to records on cause of death. A key advantage of this dataset is that individual data on socioeconomic status are available. Clegg et al. find consistent gradients in incidence rates by socioeconomic status for various forms of cancer. They find that the incidence of lung cancer and colorectal cancer was lower for individuals with higher socioeconomic status while the incidence of prostate cancer for men and breast cancer for women increased with socioeconomic status (see also [18]). For Canada, McDermott et al. [26] estimate standardized incidence rates for immigrants from many countries of birth and for most forms of cancer based on an administrative dataset that links immigrant landing records with cancer registry and mortality administrative data. Overall, immigrants tend to have lower standardized incidence rates for most types of cancer though exceptions were a relatively higher incidence of cervical, nasopharyngeal and liver cancers among East Asian immigrants. The dataset that McDermott et al. [26] uses is limited in that it includes individual data only on immigrants and also does not have individual data on socioeconomic status. (Linked datasets are more widely available in some European countries such as the Swedish Family Cancer Database, used by [12, 13, 23, 28]).

While research on population-level cancer outcomes among immigrants is limited, there is a large body of work on many different dimensions of the health of immigrants, including disease prevalence, health behaviors, and service use such as cancer screening. The general conclusion is that a healthy immigrant effect exists whereby recent immigrants are in better health (fewer chronic conditions, better self-reported health, less obesity fewer negative health behaviors) than otherwise comparable host country non-immigrants (e.g., [1, 2, 17, 25, 33], Singh and Siahpush, [39, 40]). In a systematic review of the literature on the health of immigrants to Canada, De Maio [9] finds that there is overwhelming support for the healthy immigrant effect across a range of health outcomes. Possible reasons for the healthy immigrant effect include the self-selection of immigrants who may be healthier and wealthier, differences in current and previous health behaviors such as smoking, alcohol consumption and diet, as well as differences in access to health services, the takeup of health screening, and/or differences in attitudes to health maintenance that lead to differences in the incidence of diagnosed conditions [25]. However for some measures of health this advantage dissipates over years in the host country and converges to native-born levels within 10–15 years or sooner. One limitation in much of this work is information on health outcomes is self-reported. (See also [31], who reviews data development in Canada relevant to immigrant health research). One example of recent work using linked survey-administrative data in Canada is Ng [32] who uses Canadian census data linked to vital statistics and finds a healthy immigrant effect in mortality that persists even after 20 years or more years in Canada. He also finds significant variation by country of birth and area of residence after migration (see also [43], for an examination of race/ethnicity and health in Canada).

This paper uses a large linked individual level dataset on Canadian residents to investigate differences in the incidence of cancer between immigrants and non-immigrants after controlling for a range of demographic and socioeconomic characteristics. We investigate how cancer incidence varies by country of birth, year of arrival, and time spent in Canada. In particular we are interested in examining whether there is a healthy immigrant effect in terms of the incidence of cancer and whether any gap in cancer incidence narrows with additional years in Canada. In addition, we distinguish between first generation immigrants and Canadian born residents by racial or ethnic groups. We consider the overall incidence of cancer in all sites as well as the incidence of the most common forms of cancer: prostate, colorectal and lung cancer for men, and breast, colorectal, and lung cancer for women. We also examine the robustness of the findings to controlling indirectly for differences in smoking behavior. To our knowledge, at least some parts of each question we examine have not previously been studied in Canada using a large population-based dataset.

## Methods

The data were prepared by Statistics Canada and are based on a large cohort of approximately 2.5 million individuals aged 25 or older who participated in the long form of the 1991 Census of Canada. The Census contains detailed information as of 1991 on individual education level, immigrant status, country of birth, year of arrival, ethnicity, occupation, and personal and household income from various sources. Census records on individuals in this cohort are linked to 1990–91 tax records of Canadian tax filers, Canadian Cancer Registry (CCR) data from 1984 to 2003 and the Canadian Mortality Database (CMD) from 1991 to 2008. (Statistics Canada released an updated file with cancer data to 2008 but disclosure restrictions precluded us from using the updated dataset for this project.) Overall approximately 80% of Census respondents were able to be matched to the administrative data. Wilkins et al. [45], Peters and Tjepkema [34] and Peters et al. [35] provide further discussion of the census cohort database, including details of how the datasets were linked. For each individual the CCR reports the date of diagnosis as well as the site of tumor, and each individual is linked to his or her census information. Linking to the CMD file identifies the date and cause of death for members of the census cohort who died in Canada after 1991, information necessary for our duration analysis. The Census cohort is also linked to the postal code of residence drawn from T1 tax file data. Thus, postal code of residence as of the date of filing is available each year from 1986 to 2008 as long as the individual filed a tax return in that year. This dataset was accessed through the Statistics Canada Research Data Centre Network following Statistics Canada’s standard application, approval, vetting and disclosure policies for access to data contained within the RDC network.

The focus of our analysis is the subset of the 1991 Census cohort for whom location information from the tax filer data is available and can be linked to Cancer Registry data. Since individuals must have been alive and present in Canada as of the Census day, immigrants and returning residents arriving in Canada after 1991 are not part of the Census cohort. A total of 2.7 million individuals can be linked to at least one tax return, including approximately 500,000 immigrants. There are roughly 247,000 cases of cancer diagnoses in this cohort between 1991 and 2003. Individuals diagnosed with cancer prior to 1991 are excluded.

Each individual in the sample is first diagnosed with cancer at a particular point in time or leaves the sample for other reasons before this takes place. By definition the likelihood of diagnosis at a point in time T conditional on this not taking place in the past is

$$ \underset{\triangle t->0}{ \lim}\left(\frac{ \Pr \Big( t\le T\le t+\triangle t}{\triangle t}\right)/\left(1- \Pr \left( T\le t\right)\right) $$

where Pr(*t* ≤ *T* ≤ *t* + ∆*t*) is the probability of diagnosis between a point in time t and a point in time in the future *t* + ∆*t*. Pr(*T* ≤ *t*) is the probability of being diagnosed at the point in time t or earlier and this is a failure rate. Diagnosis at a point in time conditional on this not taking place in the past is related to the dependent variable and the associated failure rates are discussed below.

The sample reports the calendar day of diagnosis and does not report the stage at which cancer is at or when it first began hence wider time spans are examined. In particular the time range that the sample reports information on is divided into 13 calendar years.

(1*Jan*1991, 31*Dec*1991] , (1*Jan*1992, 31*Dec*1992] ,  …  , (1*Jan*2003 , 31*Dec*2003].

Within each year an individual is either diagnosed with cancer for the first time, is not diagnosed with cancer and never has been, or has been diagnosed with cancer in previous years and hence is no longer included in the sample. Many individuals enter the sample on January 1st in 1991 and before this have been at risk. This topic is further discussed below.

After a person enters the sample there is an observation for each year during which he or she is not diagnosed for the first time and remains in the sample. For those who are diagnosed there is also one observation associated with the year in which this takes place. Given the relatively wide time spans discrete duration analysis is applied and a logit model is estimated [16]. In order to examine the robustness of findings to the functional form a Cox proportional hazard model is also estimated. Sueyoshi [41] shows how a logistic model with interval-specific intercepts is consistent with an underlying continuous time duration model in which the within-interval durations follow a log-logistic distribution.

Under a logit model individual i’s log odds of conditional diagnosis in year t is

1 $$ \begin{array}{l}\mathit{\ln}\left(\frac{ \Pr \left(31{Dec}_{t-1}< T\le 31{Dec}_t\right|\ {T}_i>31{Dec}_{t-1}}{1- \Pr \left(31{Dec}_{t-1}< T\le 31{Dec}_t\right|\ {T}_i>31{Dec}_{t-1}}\right)\\ {}={\beta}_0+ X{1}_{i, t}{\beta}_1+ X{2}_i{\beta}_2+{\beta}_3{urban}_{i, t}+\\ {}{\beta}_4\ { i mmigrant}_i\cdot years\  in\ {Canada}_{i, t}+{\beta}_5{ i mmigrant}_i\cdot years\  in\ {Canda}_{i, t}^2\end{array} $$

For each individual there is an observation for each January 1st to December 31st time span examined. The dependent variable is equal to one if a person is diagnosed and equal to zero otherwise. The term *X*1_*i* , *t*_ is a row vector of calendar year, birth year, and age indicators for individual i in time period t and *β*_1_ is the associated column vector of parameters. Individuals are assumed to first be at risk when they turn 25 and as mentioned above many individuals enter the sample at an older age level. For this reason and in order control for changes over calendar time, across birth cohorts, and age levels one set of indicator variables are used to account for these three factors [36]. This approach imposes very little parametric structure.

The row vector *X*2_*i*_ includes individual i’s education level in 1991 (less than high school, high school only, some post-secondary, bachelor’s degree, and higher degree), ethnicity if Canadian born (White, Black, South Asian, West Asian/Mideast, other Asian, Latin American, and other) region of birth if an immigrant (English speaking developed countries including the US, UK and Ireland, Israel and South Africa, continental Europe, Western Asia/Mideast, other Asia, and other regions), and period of arrival in Canada if an immigrant (1980–90, 1970–79, ..., 1930–39, pre-1930). We omit Canadian born aboriginal people from the sample given the complexities of Aboriginal health outcomes in Canada and consider it in other work. Table 1 provides some descriptive statistics on sample composition by region of birth for immigrants and ethnicity/race for the Canadian-born. It can be seen that even with a large sample, counts for some Canadian born ethnic groups are relatively small.

**Table 1:** Sample characteristics–immigrant region of birth and Canadian born ethnicity

	% of total sample of individuals
Immigrant	19.8
Region of Birth	
US	1.1
UK/Ireland	4.0
Continental Europe	8.2
Western Asia/Mideast	1.0
South Asia	2.9
Other Asian nations	0.6
Africa	0.4
Americas	1.7
Canadian born	80.2
Race/ethnicity	
White	79.3
Black	0.2
West Asian/Mideast	0.01
South Asian	0.1
Other Asian	0.4
Latin American	0.02
# observations	31,031,320
# individuals	2,565,100
# cancer cases	204,100

Note: UK/Ireland includes Israel and South Africa. Sample excludes Aboriginal Canadians

With a single cross section of data, arrival year and years in Canada are in fact perfectly correlated since year of arrival = current year – years in Canada. Since our dataset is longitudinal however, these effects can be separately identified since a given arrival cohort can be observed each year over the whole sample period. We prefer to proxy socioeconomic status using education in 1991 rather than income in 1991 since the former is subject to much less variation over time for adults. The other factors examined can vary over an individual’s time at risk. These include an indicator for whether the person resides in large urban region of residence (city of 500,000+ population) during the year, and for immigrants the number of years spent in Canada measured as a continuous variable and its square. Postal codes in the Census cohort dataset must be mapped to Statistics Canada geocodes in order to assign rural/urban status. Differences in cancer diagnosis between rural and urban areas can arise for a variety of reasons such as extent of exposure to pollution and are well-established in the literature (e.g., [15]).

A limitation of the Census cohort is that there is no person-level information on smoking and other health behaviors. Sanmartin et al. [37] estimate models of smoking behavior using survey data and then use the resulting estimates to predict smoking behavior of individuals in the Census cohort where the predictions are based on a set of variables common to both data sets. Identification is primarily through functional form. To increase variation in predicted smoking behavior and the closeness of the match of predicted smoking to actual but unobserved behavior, we first estimate discrete time hazard models of years until becoming a daily smoker using the retrospective information on starting smoking available in the Canadian Community Health Survey (CCHS). Logit estimations for the conditional probability of starting smoking are estimated separately for 232 distinct subpopulations based on decade turned 10 and age group, (29 categories), region of birth including Canada (4 categories), and gender (2 categories). For each subsample the regression estimated includes controls for education level, specific country of birth, province of residence in Canada, language first learned, race/visible minority status, and tobacco price. Next, using these results, the age-specific mean hazard rate is computed for a large number of subgroups based on education and region of residence in Canada as well as region of birth, gender, age and decade turned 10. These estimates are used to predict the number of people per thousand of each particular set of characteristics who have never started to smoke daily, and this prediction is merged with the cancer data for people with the same set of characteristics and included as a covariate.

We estimate these models separately for men and women, and allow for clustering of person-specific errors over time. Since the risk factors associated with particular cancers can vary widely, we also estimate eq. (1) separately for the most common cancer types: for women, we estimate the determinants of breast cancer, colorectal cancer and lung cancer; for men, we estimate the determinants of prostate cancer, colorectal cancer and lung cancer.

## Results

We begin by estimating simple Logit models separately for men and women that include age/birth cohort/time period controls, an indicator for foreign born status and a quadratic in years-since-migration (YSM). The immigration estimates are presented in Table 2. For any cancer and for the specific cancers considered and for both men and women there is clear evidence of a significant initial gap in the incidence of being diagnosed with cancer upon arrival in Canada for immigrants relative to the Canadian born, and a significant narrowing of this gap with years in Canada towards Canadian born levels of cancer incidence. As can be seen in the top panel of Table 2, for recently arrived immigrant men the odds of being diagnosed with any cancer are only about half of what they are for non-immigrant men (OR 0.517, *p*-val .000). After arrival, an additional year in Canada increases the odds of being diagnosed with cancer by 0.02 (OR 1.02, *p*-val .000) although the quadratic term in YSM reduces the magnitude of the increase as YSM gets large. A survival plot based on these results (available on request) indicates convergence and leveling off at Canadian-born levels of cancer incidence at around 50 years in Canada. Results for prostate, colorectal and lung cancer for men are remarkably consistent. In the lower panel of Table 2, the odds of recently arrived immigrant women being diagnosed with cancer are only about 64% of those for Canadian born women (OR 0.638, *p*-val .000). Odds increase with additional years in Canada but at a slower rate than for immigrant men (YSM OR 1.010, *p*-val .000; YSM^2^ OR 1.000, *p*-val .164). A survival plot (available on request) indicates convergence to Canadian born levels at around 60 years in Canada. For specific cancers, patterns are similar but the initial difference in odds varies by cancer site: OR 0.726, *p*-val .000 for breast cancer, OR 0.538, *p*-val .000 for colorectal cancer and OR 0.289, *p*-val .000 for lung cancer. Overall this provides clear preliminary evidence of a healthy immigrant effect for cancer overall and by site, and a gradual convergence to Canadian-born levels after many years in Canada. As well, adding in controls for education level, province of residence and urban/rural status does not alter this conclusion. While those other covariates are themselves usually significant, given our focus on immigrants and space limitations we do not discuss those results further in this paper but will make them available on request.

**Table 2:** Selected Logistic odds ratios of cancer incidence of adults by immigrant status and years in Canada (ysm)

	OR	*p*-val	OR	*p*-val	OR	*p*-val	OR	*p*-val
Men	Any cancer		Prostate		Colorectal		Lung	
Cdn born	1.000	--	1.000	--	1.000	--	1.000	--
Immigrant	0.517	0.000	0.472	0.000	0.477	0.000	0.427	0.000
YSM	1.020	0.000	1.020	0.000	1.020	0.000	1.020	0.000
YSM^2^	1.000	0.000	1.000	0.001	1.000	0.001	1.000	0.001
Women	Any cancer		Breast		Colorectal		Lung	
Cdn born	1.000	--	1.000	--	1.000	--	1.000	--
Immigrant	0.638	0.000	0.726	0.000	0.538	0.000	0.289	0.000
YSM	1.010	0.000	1.010	0.038	1.020	0.001	1.010	0.035
YSM^2^	1.000	0.164	1.000	0.335	1.000	0.051	1.000	0.403

Note: regressions also include controls that jointly account for age, birth cohort and calendar year. Sample excludes Aboriginal Canadians

One limitation of treating immigrants as a homogeneous group is that what appears to be convergence to Canadian born levels could instead be a lower intrinsic likelihood of being diagnosed with cancer by more recent immigrant groups. As well, determining how results might vary by immigrant dimensions such as country of birth can yield insights into what might underpin observed differences in the incidence of cancer diagnosis. We estimate Logit models with additional covariates for region of birth and period of arrival as well as additional controls for visible minority status for the Canadian born. (The high degree of correlation between visible minority group and country of birth for immigrants precludes the inclusion of both sets of covariates for immigrants). In Table 3, we present results by gender for cancer in any site while in Tables 4 and 5 we present results for specific cancer sites for men and women respectively. With this set of additional immigrant variables included, the interpretation of the immigrant indicator variable alone is the estimated difference in odds on arrival in Canada for the base group of immigrants who arrived prior to 1930 from the United States, while the estimated odds ratios for period of arrival and region of birth modify this effect. As well, with visible minority indicators included for native-born Canadians, the underlying comparison group is white Canadian born men. In Table 3 it can be seen that after controlling for other factors, cancer diagnosis varies significantly across regions of birth and time in Canada. For men, the odds ratio is significantly less than one while the odds ratio for years in Canada is significantly greater than one. Together these results indicate a significant healthy immigrant effect for men but a narrowing of the advantage with time spent in Canada. The odds ratios for the arrival period cohort effects are all less than one but not significant. Region of birth appears to matter much more, with immigrants from most regions significantly less likely to be diagnosed with cancer in a given year relative to immigrants from the US. For example, the odds of a cancer diagnosis for an immigrant man from South Asia are only half that of an immigrant man from the US (OR 0.494 *p*-val .000). Even immigrant men from continental Europe have significantly lower odds of being diagnosed with cancer than immigrants from the US (OR 0.905, *p*-val .001). The only exception is immigrant men from UK/Ireland whose odds ratio is not significantly different from US born men. It should be emphasized that since US men themselves are significantly less likely than Canadian born white men, the magnitude of the differences between most immigrants and Canadian born white men are even greater. Canadian born men who identify as a visible minority show marked variation in the likelihood of being diagnosed with cancer. Canadian born men of Middle Eastern descent have higher odds of being diagnosed with cancer (OR 1.231, *p*-val .015), while black and Latin American men are not significantly different from white men, and South Asian men and other Asian men are significantly less likely to be diagnosed with cancer (OR 0.530, *p*-val .024 and OR 0.758, *p*-val .000 respectively).

**Table 3:** Selected Logistic odds ratios by demographic and socioeconomic characteristics of adults (any cancer)

	MEN		WOMEN	
	OR	*p*-val	OR	*p*-val
Race/ethnicity-Cdn born				
White	1.000	--	1.000	--
Black	1.134	0.101	0.822	0.023
West Asian/Mideast	1.231	0.015	0.959	0.666
South Asian	0.530	0.024	0.851	0.497
Other Asian	0.758	0.000	0.940	0.294
Latin American	0.600	0.376	0.903	0.792
Immigrant	0.723	0.006	1.174	0.231
Period of Arrival				
Arrived 1980–91	0.935	0.491	0.746	0.014
Arrived 1970–79	0.931	0.431	0.755	0.012
Arrived 1960–69	0.904	0.227	0.746	0.005
Arrived 1950–59	0.891	0.114	0.789	0.010
Arrived 1940–49	0.906	0.144	0.883	0.126
Arrived 1930–39	0.945	0.311	0.773	0.000
Arrived pre 1930	1.000	--	1.000	--
Years in Canada (YSM)	1.015	0.000	1.006	0.152
YSM^2^	1.000	0.006	1.000	0.101
Region of Birth				
US	1.000	--	1.000	--
UK/Ireland	1.049	0.140	0.993	0.831
Other Europe	0.905	0.001	0.820	0.000
Western Asia/Mideast	0.753	0.000	0.799	0.002
South Asia	0.494	0.000	0.588	0.000
Other Asian nations	0.714	0.000	0.726	0.000
Africa	0.763	0.000	0.790	0.000
Americas	0.891	0.007	0.671	0.000
# observations	15,403,340		15,627,980	
# individuals	1,286,260		1,278,840	
# cancer cases	110,300		93,800	

Note: UK/Ireland includes Israel and South Africa. Regression includes age/year/birth cohort effects, province, marital status, urban/rural residence, and educational attainment. Sample excludes Aboriginal Canadians

**Table 4:** Logistic odds ratios by demographic and socioeconomic characteristics of adult men

	Prostate		Lung		Colorectal	
	OR	*p*-val	OR	*p*-val	OR	*p*-val
Race/ethnicity-Cdn born						
White	1	--	1	--	1	--
Black	1.498	0.003	1.116	0.561	0.855	0.521
West Asian/Mideast	0.699	0.094	1.509	0.030	1.596	0.021
South Asian	n/a		0.261	0.180	0.630	0.514
Other Asian	0.769	0.013	0.507	0.000	0.956	0.742
Latin American	n/a		n/a		1.594	0.646
Immigrant	0.397	0.000	0.945	0.851	1.205	0.555
Period of Arrival						
Arrived 1980–91	1.078	0.673	1.060	0.816	0.458	0.003
Arrived 1970–79	0.966	0.826	1.117	0.631	0.561	0.018
Arrived 1960–69	0.818	0.163	1.279	0.250	0.594	0.022
Arrived 1950–59	0.783	0.051	1.279	0.190	0.608	0.013
Arrived 1940–49	0.818	0.084	1.205	0.286	0.717	0.070
Arrived 1930–39	0.933	0.464	1.224	0.155	0.727	0.037
Arrived pre 1930	1	--	1	--	1	--
Years in Canada (YSM)	1.044	0.000	0.985	0.126	1.010	0.385
YSM^2^	1.000	0.000	1.000	0.067	1.000	0.160
Region of Birth						
US	1	--	1	--	1	--
UK/Ireland	1.059	0.324	1.008	0.923	1.275	0.010
Other Europe	0.860	0.008	0.842	0.041	1.190	0.058
Western Asia/Mideast	0.851	0.206	0.576	0.005	0.922	0.669
South Asia	0.670	0.000	0.273	0.000	0.462	0.000
Other Asian nations	0.506	0.000	0.696	0.001	1.064	0.571
Africa	0.908	0.354	0.532	0.000	0.655	0.021
Americas	1.588	0.000	0.419	0.000	0.814	0.110
# observations	13,321,560		15,398,640		15,403,340	
# individuals	1,275,250		1,285,860		1,286,260	
# cancer cases	29,620		18,110		14,940	

Note: as for Table 3

**Table 5:** Logistic odds ratios by demographic and socioeconomic characteristics of adult women

	Breast		Colorectal		Lung	
	OR	*p*-val	OR	*p*-val	OR	*p*-val
Race/ethnicity-Cdn born						
White	1	--	1	--	1	--
Black	0.823	0.210	0.716	0.268	0.705	0.226
West Asian/Mideast	1.169	0.318	0.868	0.656	1.160	0.594
South Asian	1.371	0.346	n/a		1.125	0.868
Other Asian	1.144	0.159	0.996	0.980	0.448	0.002
Latin American	1.290	0.663	n/a		1.557	0.663
Immigrant	1.186	0.474	0.687	0.346	2.118	0.113
Period of Arrival	1	--	1	--	1	--
Arrived 1980–91	0.702	0.108	1.097	0.779	0.411	0.020
Arrived 1970–79	0.707	0.099	1.077	0.805	0.469	0.025
Arrived 1960–69	0.690	0.062	1.017	0.952	0.505	0.027
Arrived 1950–59	0.676	0.025	0.988	0.960	0.747	0.278
Arrived 1940–49	0.719	0.035	1.039	0.860	0.933	0.768
Arrived 1930–39	0.822	0.158	0.441	0.000	0.907	0.614
Arrived pre 1930	1	--	1	--	1	--
Years in Canada (YSM)	1.013	0.062	1.012	0.349	0.984	0.286
YSM^2^	1.000	0.040	1.000	0.582	1.000	0.392
Region of Birth						
US	1	--	1	--	1	--
UK/Ireland	0.984	0.768	1.008	0.937	1.063	0.550
Other Europe	0.824	0.000	0.928	0.427	0.450	0.000
Western Asia/Mideast	1.059	0.614	0.544	0.033	0.295	0.002
South Asia	0.658	0.000	0.424	0.000	0.180	0.000
Other Asian nations	0.737	0.000	0.765	0.026	0.590	0.000
Africa	0.986	0.879	0.481	0.003	0.390	0.001
Americas	0.716	0.000	0.671	0.003	0.266	0.000
# observations	15,627,980		15,620,350		15,570,080	
# individuals	1,278,840		1,278,220		1,278,840	
# cancer cases	29,190		10,360		9990	

Note: as for Table 3

For women, the base group of immigrants from the US who arrived prior to 1930 are not significantly different from Canadian born white women in their odds of being diagnosed with cancer, but for every arrival cohort after that the odds are significantly lower than for the base category. As well, immigrant women from every region of birth except UK/Ireland have significantly lower odds of cancer diagnosis than immigrants from the US. The YSM terms are also not significant, implying no change in cancer diagnosis for immigrant women with years in Canada. In sum, the results together indicate that for most immigrant women – those arriving after 1930 and those from countries other than the US and UK/Ireland – the odds of being diagnosed with any cancer are significantly lower than for Canadian born white women but there is little evidence of convergence over time to Canadian born levels. For women, the odds ratios for immigrants and years in Canada are not significant although later arrivals have significantly lower odds of being diagnosed with cancer. For Canadian born visible minority women, all minority groups have lower estimated odds of being diagnosed with cancer than white women but the result is only significant for black women.

In Table 4, we report odds ratios for men for three types of cancer: prostate, colorectal and lung. For Canadian born minorities, small sample sizes preclude estimation for some groups, especially for prostate cancer. The results indicate a significantly higher likelihood of prostate cancer for black Canadian born men (OR 1.498, *p*-val .003), a result consistent with the US literature. For Canadian born men of other Asian ethnicities, the odds of being diagnosed with prostate cancer are lower than they are for white men (OR 0.769, *p*-val .013). Canadian born men of West Asian/Mideast ethnicity have higher odds of colorectal cancer (OR 1.596, *p*-val .021) and for lung cancer (OR 1.509, *p*-val .030) than white men while other Asian men have lower odds of lung cancer (OR 0.507, *p*-val .000).

For US born immigrants who arrived prior to 1930 (our base category), their odds of prostate cancer are significantly lower than for non-immigrants on arrival (OR 0.397, *p*-val .000) but increase with years in Canada (YSM OR 1.044, *p*-val .000; YSMsq OR 1.000, *p*-val .000). Arrival period cohort effects are mostly less than one but none is significant at the 5% level. There are however very large differences in the odds of being diagnosed with prostate cancer by country of birth. Relative to US born immigrants (who themselves are significantly less likely to be diagnosed with prostate cancer than Canadian born whites), estimates for OR range from a low of 0.506 (*p*-val .000) for other Asian immigrants and 0.670 (*p*-val .000) for immigrants from South Asia to a high of 1.588 (*p*-val .000) for immigrants from the Americas. It should be noted that the net effect for this group is that their odds of being diagnosed with prostate cancer relative to Canadian-born whites is still significantly less than 1 (results on request). For colorectal cancer, the odds ratio for the base group of US born immigrants arriving prior to 1930 is not significantly different from that of Canadian born white men. However, relative to these early arrivals almost all immigrant cohorts arriving after 1930 have significantly lower odds of diagnosis, with the difference being more pronounced for more recent cohorts. The net effect is that for immigrants from the US arriving after 1949, their odds of colorectal cancer diagnosis are significantly lower than those of Canadian born whites (available on request). As with prostate cancer there is marked variation in diagnosis with colorectal cancer by place of birth. Immigrants from the UK/Ireland have higher odds of a colorectal cancer diagnosis than immigrants from the US (OR 1.275, *p*-val .010), offsetting the lower likelihood experienced by most arrival cohorts. Immigrants from South Asia and Africa have lower odds of colorectal cancer diagnosis than US born immigrants (OR 0.462, *p*-val .000 and OR 0.655, *p*-val .021 respectively) while for others the estimated odds ratio is not significantly different from 1.

For lung cancer, there is no significant difference in the odds of a US born male being diagnosed compared to a Canadian-born white male, nor are any of the arrival period effects and years since migration effects significant. However there are again marked differences by country of birth, with the odds ratio for every region except UK/Ireland significantly less than 1. This implies then that for immigrant men born elsewhere than the US or UK/Ireland the odds of being diagnosed with lung cancer are significantly lower than for Canadian born white men.

Table 4 presents results for women for three cancer sites: breast, colorectal and lung. There are almost no significant differences in the odds of being diagnosed with any of these cancers for Canadian-born visible minority women compared to white women, possibly because of relatively small sample sizes for these groups. The exception is that for lung cancer Canadian-born ‘other Asian’ women have a significantly lower odds of cancer diagnosis than Canadian-born white women (OR 0.448, *p*-val .002).

For the reference group of immigrant women born in the US who arrived prior to 1930, there is no significant difference in the odds of cancer diagnosis for each of the cancers considered. Period of arrival in Canada is a significant determinant of breast cancer diagnosis and lung cancer diagnosis, with many more recent arrival groups significantly less likely to be diagnosed with cancer than the earliest arrivals. Only for breast cancer are there changes in incidence with years in Canada, with the net effect a slow increase over time. For region of birth, the general pattern again is that there is significant variation by birth region but overall the pattern is of lower likelihood of a cancer diagnosis for each cancer site. Immigrant women from the UK and Ireland have essentially the same odds of being diagnosed with any of the three forms of cancer as immigrant women from the US (and so by extension Canadian born women given the insignificant base group estimate). Immigrant women from continental Europe, South Asia, other Asia and the Americas have significantly lower odds of being diagnosed with breast cancer, with estimated odds ratios 0.824 (*p*-val .000), 0.658 (*p*-val .000), 0.737 (*p*-val .000), and 0.716 (*p*-val .000) respectively. For colorectal cancer, immigrant women from every region except continental Europe and UK/Ireland have significantly lower odds of cancer diagnosis than the base group of immigrant women born in the US. Similarly, immigrant women from every region except the UK/Ireland have lower odds of being diagnosed with lung cancer.

## Extension - Smoking

One potentially important set of confounding factors is health behaviors such as smoking that are major risk factors for many types of cancer. It is well known that smoking rates vary inversely with education levels but it is also established in the literature that immigrants on average are significantly less likely to smoke than comparable non-immigrants but with marked variation by country of birth, gender and time spent in the host country [17, 20, 29]. One of the major limitations of the Census-cohort (and indeed almost all administrative datasets) is the lack of information on health behaviors. Without controls for differences in health behavior, it may be that at least some of the effect of immigrant status on the likelihood of being diagnosed with cancer is in reflecting these differences. In order to address this, we rely on survey data from Statistics Canada’s Canadian Community Health Surveys.

Selected results are reported in Table 6 for men and Table 7 for women. The predicted smoking measure itself is strongly significant for cancer overall (OR 0.958 *p*-val .000 for men, OR 0.945 *p*-val .000 for women) as well as for prostate cancer (OR 0.946 *p*-val .004) and lung cancers (OR 0.906 *p*-val .000 for men, OR 0.854 *p*-val .000 for women), indicating that the higher the probability of an individual never becoming a daily smoker, the lower is the likelihood of a cancer diagnosis. Of more interest is that including this variable in our duration models does not have a large quantitative effect on the estimates of the immigrant controls. Comparing results with and without smoking controls indicates that while in many cases odds ratios with the smoking controls are marginally closer to 1, they remain significant except for female immigrants from Africa and Western Asia for any cancer and for lung cancer. Overall, our estimates of the healthy immigrant effect for cancer diagnosis are not due to group-level differences in smoking behavior.

**Table 6:** Logistic odds ratios by demographic and socioeconomic characteristics of adult men, with smoking controls

	Any cancer		Prostate		Colorectal		Lung	
	OR	*p*-value	OR	*p*-value	OR	*p*-value	OR	*p*-value
Immigrant	0.750	0.015	0.389	0.000	1.258	0.470	1.093	0.767
Period of Arrival								
Arrived 1980–91	0.937	0.507	1.096	0.610	0.442	0.002	1.049	0.849
Arrived 1970–79	0.935	0.457	0.984	0.916	0.540	0.012	1.107	0.662
Arrived 1960–69	0.907	0.245	0.845	0.244	0.566	0.013	1.247	0.303
Arrived 1950–59	0.891	0.116	0.796	0.069	0.581	0.007	1.258	0.224
Arrived 1940–49	0.905	0.140	0.829	0.106	0.687	0.041	1.191	0.319
Arrived 1930–39	0.942	0.286	0.939	0.505	0.715	0.028	1.212	0.178
Arrived pre 1930	1.000		1.000		1.000		1.000	
Years in Canada (YSM)	1.015	0.000	1.044	0.000	1.010	0.371	0.985	0.128
YSM^2^	1.000	0.007	1.000	0.000	1.000	0.146	1.000	0.064
Region of Birth								
US	1.000		1.000		1.000		1.000	
UK/Ireland	1.017	0.603	1.004	0.948	1.265	0.014	0.963	0.669
Other Europe	0.850	0.000	0.805	0.000	1.178	0.093	0.722	0.000
Western Asia/Mideast	0.777	0.000	0.971	0.821	0.891	0.548	0.575	0.006
South Asia	0.503	0.000	0.673	0.000	0.458	0.000	0.299	0.000
Other Asian nations	0.728	0.000	0.507	0.000	1.071	0.540	0.768	0.015
Africa	0.790	0.000	1.042	0.694	0.642	0.017	0.538	0.001
Americas								
10% increase in probability of never starting to smoke	0.958	0.000	0.946	0.004	1.007	0.805	0.906	0.000

Note: As for Table 3. Regressions also include controls for ethnicity of Canadian-born

**Table 7:** Logistic odds ratios by demographic and socioeconomic characteristics of adult women, with smoking controls

	Any cancer		Breast		Colorectal		Lung	
	OR	*p*-value	OR	*p*-value	OR	*p*-value	OR	*p*-value
Immigrant	1.202	0.170	1.190	0.469	0.673	0.322	2.389	0.067
Period of Arrival								
Arrived 1980–91	0.749	0.010	0.702	0.093	1.082	0.795	0.452	0.020
Arrived 1970–79	0.738	0.011	0.698	0.103	1.097	0.781	0.397	0.016
Arrived 1960–69	0.746	0.005	0.686	0.059	1.016	0.955	0.480	0.018
Arrived 1950–59	0.786	0.009	0.671	0.022	0.990	0.967	0.719	0.224
Arrived 1940–49	0.880	0.117	0.712	0.030	1.037	0.864	0.919	0.722
Arrived 1930–39	0.784	0.001	0.826	0.168	0.438	0.000	0.919	0.662
Arrived pre 1930	1.000		1.000		1.000		1.000	
Years in Canada (YSM)	1.006	0.118	1.014	0.052	1.011	0.385	0.985	0.296
YSM^2^	1.000	0.085	1.000	0.035	1.000	0.613	1.000	0.391
Region of Birth								
US	1.000		1.000		1.000		1.000	
UK/Ireland	0.984	0.618	0.973	0.625	0.990	0.916	1.008	0.938
Other Europe	0.879	0.000	0.844	0.003	0.883	0.206	0.524	0.000
Western Asia/Mideast	1.015	0.854	1.171	0.224	0.483	0.016	0.527	0.123
South Asia	0.715	0.000	0.706	0.002	0.363	0.000	0.312	0.001
Other Asian nations	0.878	0.012	0.788	0.009	0.656	0.008	1.031	0.876
Africa	0.991	0.894	1.083	0.483	0.434	0.002	0.701	0.267
Americas	0.848	0.003	0.787	0.012	0.576	0.001	0.467	0.001
10% increase in probability of never starting to smoke	0.945	0.000	0.976	0.162	1.039	0.169	0.854	0.000

Note: As for Table 3. Regressions also include controls for ethnicity of Canadian-born

## Extension - Continuous Time Models

We estimate continuous time Cox proportional hazard models that allow for age, location, and years since migration for immigrants to be time varying while other controls are treated as time invariant. Results for both education controls and immigrant controls are comparable in terms of the estimated relationship between these variables and the diagnosis of cancer. We also implement an indirect adjustment method to control for unobserved smoking [38] and it makes little difference to the results (these are not reported but are available on request).

## Discussion

This is one of the first papers to examine whether there is a healthy immigrant effect in terms of cancer incidence and if so, whether any advantage is lost over time spent in the host country. We are able to account for differences in personal socioeconomic status as measured by the highest level of educational attainment, and we allow for variation in outcomes by region of birth, period of arrival and time in Canada. We also examine the extent to which differences in cancer outcomes by region of origin persist among the Canadian-born descendants of different ethnic groups. Consistent with the literature for other measures of health (e.g., [2, 9, 25]) using a simple set of immigrant controls we find strong evidence that there is a healthy immigrant effect for immigrant men and women after controlling for other factors. We also find that this advantage narrows with years in Canada, converging after around 50 years in the host country. However, allowing differences by immigrant characteristics, including region of birth, year of arrival in Canada and years spent in Canada reveals marked differences across immigrant groups. For immigrant men, the healthy immigrant effect plus convergence over time is evident for any cancer diagnosis and for prostate cancer diagnosis but not for colorectal or lung cancers. For colorectal cancer, arrival period effects indicate that immigrant men arriving after 1930 also have a significantly lower likelihood of a cancer diagnosis. Across regions of birth however differences are most pronounced. For example, immigrant men from South Asia have a markedly lower likelihood of any cancer diagnosis or of prostate, colorectal or lung cancer than immigrants from the US and UK/Ireland as well as Canadian born white men. Immigrant men from Africa have lower likelihood of any cancer and of colorectal and lung compared to US born whites but not prostate cancer. McDermott et al. [26] also finds broadly similar results in terms of their estimates of age-standardized incidence rates by cancer site. One possible explanation for lower might be that the rapid uptake of PSA prostate cancer screening through the 1990s (Dickinson et al. [10]) was not shared by particular immigrant groups so that the differences in prostate cancer diagnosis reflect differences in screening rather than in actual disease incidence. For immigrant women, the base group of US-born immigrants who arrived prior to 1930 are not significantly different in terms of cancer diagnosis, and it is only for breast cancer where there is some evidence of an increase with years since migration. It may be that increased use of regular cancer screening with additional time in Canada is leading to this effect. For example, Vahebi et al. [42] find mammography screening rates lower among new and recent immigrants to Ontario. However any arrival period effects or country of birth effects that are significant are uniformly less than 1, implying lower likelihood of being diagnosed with cancer for most female immigrant subgroups. For example, immigrant women from South Asia are significantly less likely than US arrivals to be diagnosed with any cancer and with breast, colorectal and lung cancers. This is true of immigrant women from continental Europe except for colorectal cancer and other Asia except for lung cancer. Persistence in lower occurrence of certain cancers among immigrants from mainly developing countries even after many years in Canada provides useful guidance as to continued research on what it is about these individuals that seems protective of cancer. Importantly, the results in most cases are not accounted for by group-level differences in lifetime smoking behavior even though smoking rates for immigrants, even from countries with relatively high smoking rates, are significantly lower than for non-immigrants [17, 29].

For second and earlier generation Canadians who belong to a visible minority, many of the estimated odds ratios are insignificant, implying no difference in the incidence of cancer diagnosis with Canadian born whites. For some groups though there is a lower likelihood of being diagnosed with cancer. Canadian born men of Middle Eastern, south Asian or other Asian descent are less likely to be diagnosed with any cancer and the same is true of Canadian born black women. For lung cancer, other Canadian born Asian men and women have a lower incidence of diagnosis. This suggests that possible barriers to timely cancer screening such as language difficulties or unfamiliarity with the healthcare system experienced by some immigrants do not account for estimated differences as these are not factors for individuals born and raised in Canada. For other groups though the likelihood of cancer diagnosis is higher than for Canadian-born whites, such as lung cancer for Middle Eastern men and Prostate cancer for black men.

A number of limitations to this work should be noted. First, although we have endeavored to control indirectly for group-level lifetime smoking patterns, individual smoking behavior is unknown. Other health behaviors such as diet and alcohol could also be incorporated from survey data although they typically are as of the survey year only. Second, useful information on stage at diagnosis is not available in the dataset for most cancers and for most of the sample period, which would help to identify whether differences are in the timing of diagnosis or actual incidence. Third, even with the large sample size, the number of cases of a specific cancer for a specific subgroup may still be insufficient to yield precise estimates, particularly for ethnic subgroups of Canadian born residents. Fourth, while we have endeavored to broadly match immigrant regions of birth and Canadian born visible minority groups for illustration, direct comparisons across generations should not be made owing to the fact that self-reported visible minority status may be open to more subjective interpretation than one’s country of birth. Nevertheless, differences in the likelihood of the diagnosis of certain cancers between immigrants and non-immigrants and between different immigrant groups based on year of arrival, time in Canada and especially country of birth are instructive in guiding future research into what it is about immigrant status that matters for cancer incidence. More broadly, expanding the immigrant health literature to include consideration of validated health conditions from administrative data removes bias that may arise from differences in how individuals self-report such conditions.

## Conclusions

This paper finds that immigrants to Canada have a significantly lower likelihood of being diagnosed with cancer, both overall and for particular sites, than for otherwise comparable non-immigrant Canadians. There are marked differences in cancer diagnosis by immigrant region of birth, with immigrants from developing regions of the world experiencing the lowest rates of cancer diagnosis. This healthy immigrant effect for cancer declines with additional years in Canada for immigrant men and women overall but differentiating immigrant groups by period of arrival and country of birth weakens this conclusion, especially for women. For many immigrant groups and for many of the cancer sites considered the gap remains even for long established immigrants. For Canadian born visible minority groups, after controlling for other covariates there are few differences though for some groups a lower likelihood of being diagnosed with certain cancers is present. The results of this paper will help guide further research on identifying the particular channels through which socio-demographic and economic characteristics drive cancer incidence.

## Abbreviations

CCHS: Canadian community health survey
CCR: Canadian cancer registry
CMD: Canadian mortality database
SEER: Surveillance, epidemiology and end results program
